# Effect of denture cleansing agents on tensile and shear bond strengths of soft liners to acrylic denture base

**DOI:** 10.15171/joddd.2017.033

**Published:** 2017-09-20

**Authors:** Farhang Mahboub, Fariba Salehsaber, Fereydoon Parnia, Vahedeh Gharekhani, Yousef Kananizadeh, Mahsa Taghizadeh

**Affiliations:** ^1^Dental and Periodontal Research Center, Tabriz University of Medical Sciences, Tabriz, Iran; ^2^Department of Prosthodontics, Faculty of Dentistry, Tabriz University of Medical Sciences, Tabriz, Iran; ^3^Department of Oral and Maxillofacial Surgery, Faculty of Dentistry, Tabriz University of Medical Sciences, Tabriz, Iran

**Keywords:** Denture cleansers, soft liners, shear bond strength, tensile bond strength

## Abstract

***Background.*** The aim of the present study was to evaluate the effect of Corega and 2.5% sodium hypochlorite cleansing agents on the shear and tensile bond strengths of GC soft liner to denture base.

***
Methods.*** A total of 144 samples (72 samples for tensile and 72 for shear bond strength evaluations) were prepared. The samples in each group were subdivided into three subgroups in terms of the cleansing agent used (2.5% sodium hypochlorite, Corega and distilled water [control group]). All the samples were stored in distilled water, during which each sample was immersed for 15 minutes daily in sodium hypochlorite or Corega solutions. After 20 days the tensile and shear bond strengths were determined using a universal testing machine. In addition, a stereomicroscope was used to evaluate fracture modes. Data were analyzed with one-way ANOVA, using SPSS 16.

***
Results.*** The results of post hoc Tukey tests showed significant differences in the mean tensile and shear bond strength values between the sodium hypochlorite group with Corega and control groups (P=0.001 for comparison of tensile bond strengths between the sodium hypochlorite and control groups, and P<0.001 for the comparison of tensile bond strengths between the sodium hypochlorite and Corega groups and the shear bond strengths between the sodium hypochlorite and Corega groups, and sodium hypochlorite and control groups).The majority of failures were cohesive in the control and Corega groups and cohesive/adhesive in the sodium hypochlorite group.

***
Conclusion.*** Immersion of soft liners in Corega will result in longevity of soft liners compared to immersion in sodium hypochlorite solution and sodium hypochlorite solution significantly decreased the tensile and shear bond strengths compared to the control and Corega groups.

## Introduction

Long-term soft liners are a group of polymeric agents with their longevity being at least 4 weeks in oral cavity, and they can be practically used for several months and even several years.^1^

Soft liners are classified into long-term and short-term types. The commercial soft liners that are currently available consist of acrylic-based long-term soft denture linings (ALTSDLs), silicone-based long-term soft denture linings (SLTSDLs) and other polymeric materials that might be heat-cured or self-cured.^1,2^

These materials are used as therapeutic materials in patients that cannot tolerate stresses resulting from dentures due to sharp, thin and severity resorbed ridges, visibility of the inferior alveolar nerve from underneath the mucosa, congenital and acquired palatal defects and severe bony undercuts. These liners distribute functional and parafunctional stresses due to their elastic properties and serve as shock absorbers.^3,4^ It has been reported that it is easier to use dentures with soft liners compared to rigid acrylic dentures. Use of these dentures is associated with significant improvements in articulation, masticatory efficacy, retention and stability of dentures, a decrease in pain perception and oral ulcers beneath the dentures and an increase in comfort and the duration of denture use.^5-7^

Brushing of these materials is not recommended due to the damage inflicted on the structure of the soft liner, and immersion in chemical agents is suggested, especially in the elderly and in patients with physical handicaps.^8,9^ When these materials are immersed in water, two reactions occur. Plasticizers and other soluble agents enter water, or the polymer absorbs water, gradually resulting in changes in the mechanical and physical properties of the materials in the oral cavity. The extrusion of the plasticizer is associated with the loss of elasticity and changes in the viscoelastic properties of the material, resulting in the rigidity and brittleness of the material and loss of the bond strength.^9-12^ On the other hand, it increases the roughness of the soft liner, accumulation of plaque and colonization of *Candida albicans*.^13^

Since one of the most important clinical challenges of prostheses is detachment of soft liners from the denture base,^14^ the present study was undertaken to evaluate shear and tensile bond strengths of soft liners bonded to acrylic dentures after immersion in different disinfecting agents and artificial saliva in an attempt to select a proper disinfecting agent to prevent or decrease changes induced by these agents.^15^

Since the forces that are clinically applied to soft liners are predominantly of the shear type, shear bond strength test is a proper technique for determining the bond strength of soft liners. On the other hand, although the tensile bond strength test does not simulate the forces applied to the soft liners in the clinic, Smith, Bates and Fowler reported that the tensile bond strength test is a proper technique for the evaluation of the bond strength of soft liners because tensile failure does not occur only due to the tensile forces; rather, some shearing forces, too, are applied during this test.^16^ In the present study, both tensile and shear bond strength tests were used to evaluate the bond strength of soft liners.

## Methods

In the present study, the shear and tensile bond strengths of GC soft liner (GC Corporation, Tokyo) bonded to Triplex Hot acrylic resin (Ivoclar Vivadent, Liechtenstein) was evaluated after immersion in Corega cleansing agent (Rossendarman Co.) and 2.5% sodium hypochlorite solution. A total of 144 samples (72 samples for shear bond strength test and 72 for tensile bond strength test) were prepared. Both groups were subdivided into 3 groups (n=24) (one of which was the control group) in terms of the cleansing agent used. The samples consisted of 2 acrylic blocks, measuring 40×10×10 mm, bonded with the use of a soft liner, measuring 10×10×3 mm.

**Figure 1:**
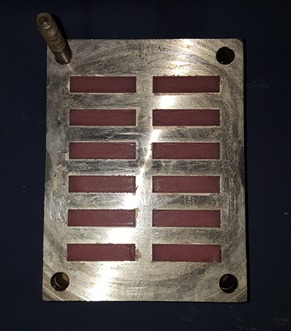
Special mold for manufacturing the acrylic blocks.

**Figure 2:**
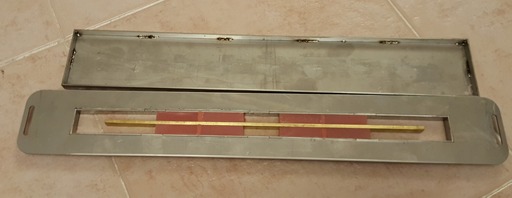
The placement of the samples in the mold for both the tensile bond strength tests.

**Figure 3:**
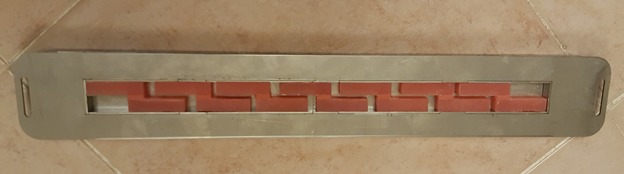
The placement of the samples in the mold for the shear bond strength tests.

**Figure 4:**
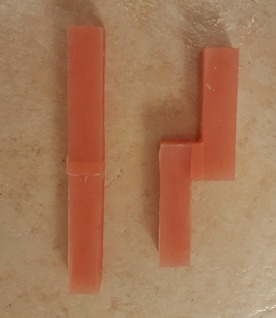
The model on the right for shear bond strength test and the model on the left for tensile bond strength test.

A special mold was designed for manufacturing the acrylic blocks. The acrylic resin was packed into the mold after mixing (Figure 1). After curing procedures, the acrylic blocks were polished, rinsed with soap and water and dried. Then the special primer of the soft liner was applied to the bonding surface with the use of a clean and dry microbrush. Then the blocks were placed in a different mold and bonded to each other two by two (Figures 2-4).

The samples were immersed in distilled water after they were prepared. One-third of the samples was immersed in 2.5% sodium hypochlorite solution once a day for 15 minutes and another one-third in Corega solution for the same period. This continued for 20 days. The remaining one-third of the samples was considered the control group.

This protocol was designed similar to the daily use of dentures by the patients.

After 20 days, a universal testing machine (UTM-Hounsfield, H5KS, England) was used for tensile and shear bond strength tests with the use of a 500-kg load cell at a strain rate of 5 mm/min, a similar method as described in previous studies.^2,9^ The force was applied until the bond failure. The maximum tensile and shear bond strength values were recorded. In addition, the failure modes were evaluated under a stereomicroscope (Nikon LV-TV) at ×10 to determine the failure mode in each sample: adhesive, cohesive and mixed.

Data were analyzed with one-way ANOVA and descriptive statistics (means ± standard deviations), using SPSS 16.0 (SPSS Inc., Chicago, USA). Statistical significance was set at P<0.05. Post hoc Tukey tests were used for two-by-two comparisons of the groups.

## Results

One-way ANOVA showed significant differences in the means of shear and tensile bond strengths between the three study groups (shear F_(2,69)_ =13.95, P<0.001; tensile: F_(2,69)_ =13.43, P<0.001).

**Figure 5:**
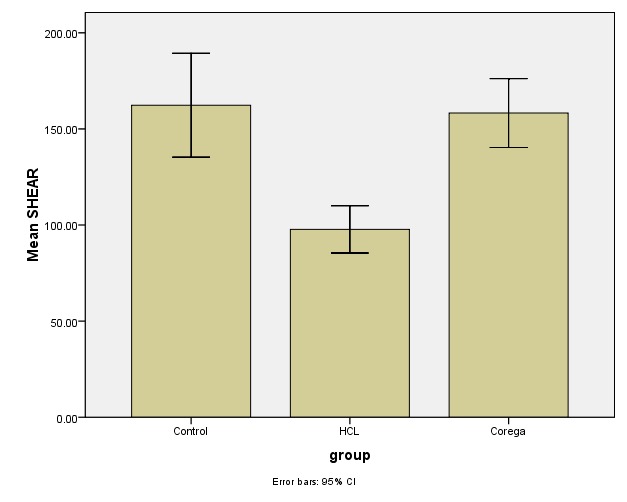
The results of one-way ANOVA comparing the shear bond strengths in three groups.

**Figure 6:**
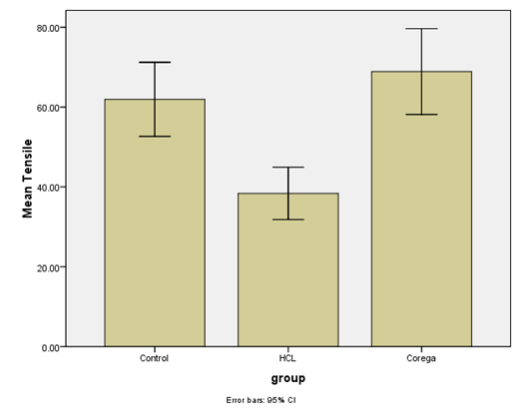
The results of one-way ANOVA comparing the tensile bond strengths in three groups.

Table 1  and 2 and Figures 5 and 6 present the descriptive statistics and the results of one-way ANOVA for comparison of the tensile and shear bond strengths.

**Table 1:** The mean shear bond strength values

**Denture cleanser**	**Mean and Std. deviation**	**Minimum‒Maximum**	**P-value**
**Control**	162.31±64.01	71.9‒276	<0.001
**Corega**	158.25±42.45	66.3‒265.6	
**HCL**	97.74±29.12	52‒151	

**Table 2:** The mean tensile bond strength values

**Denture cleanser**	**Mean and Std. deviation**	**Minimum‒Maximum**	**P-value**
**Control**	**61.93±21.98**	**40.2‒132.6**	**HCL with Control P=0.001** **HCL with Corega P<0.001**
**Corega**	**68.89±25.43**	**34.5‒123**	
**HCL**	**38.36±15.53**	**22‒86.7**	

Post hoc Tukey tests showed significant differences in the means of shear and tensile bond strengths between the sodium hypochlorite group and Corega and control groups (P=0.001 for comparison of tensile bond strengths between the sodium hypochlorite and control groups, and P<0.001 for comparison of tensile bond strengths between the sodium hypochlorite and Corega and shear bond strength between the sodium hypochlorite and Corega groups, and between the sodium hypochlorite and control groups). Therefore, it was concluded that sodium hypochlorite solution significantly decreased the shear and tensile bond strengths compared to the control and Corega groups. Shear and tensile bond strengths of Corega and control groups exhibited no significant differences.

Table 3 presents the results of failure mode evaluations in the three study groups. The majority of fractures in the control and Corega groups were cohesive, with cohesive/adhesive failures in the sodium hypochlorite group.

**Table 3:** failure mode evaluations in the three study groups

	**Denture cleanser**	**Cohesive (%)**	**Cohesive/Adhesive (%)**	**Adhesive (%)**
**Shear** **N=24**	**Control**	**75**	**25**	**0**
	**Corega**	**79.1**	**20.8**	**0**
	**HCL**	**37.5**	**58.3**	**4.16**
**Tensile** **N=24**	**Control**	**83.3**	**12.5**	**4.16**
	**Corega**	**83.3**	**16.66**	**0**
	**HCL**	**25**	**70.83**	**4.16**

## Discussion

Soft liners are added to the inner surface of dentures for homogenous distribution of force and also to decrease point pressures. Due to the damages inflicted by brushing on the structure of soft liners, immersion in chemical cleansing agents is recommended to clean the soft liners. Therefore, it is necessary to select a proper cleansing agent to prevent or decrease changes resulting from these agents. Therefore, the present study was undertaken to evaluate the bond strength of soft liners to heat-cured acrylic resins after immersion of these liners in different cleansing agents.

Based on the results, sodium hypochlorite solution decreased the shear and tensile bond strengths significantly compared to the control and Corega groups; however, Corega did not result in a significant decrease in tensile and shear bond strengths compared to the control group. The results of this study on the effect of sodium hypochlorite solution on tensile bond strengths of soft liners are consistent with those of a study by Narwal,^17^ who evaluated the effects of two cleansing agents containing sodium hypochlorite and sodium perborate on tensile bond strength and hardness of soft liners. The results showed that both materials resulted in an increase in hardness and a decrease in tensile bond strength of soft liners.

In a study by Renata et al^2^ (2003), the effects of immersion of soft liners in water and Polident at certain intervals on tensile bond strengths were evaluated. The results showed that Polident did not result in an obvious change in tensile bond strength compared to water at the intervals evaluated. Since the chemical composition of Polident and Corega are almost the same, it can be claimed that the results of the present study in relation to the lack of the effect of Corega on tensile bond strength in comparison to water are consistent with those of the study above.

In addition, Mese et al^8^ (2006) evaluated the effect of water and Polident on the tensile bond strength of different types of soft liners. The results were consistent with the results reported by Renata^2^. Yakinoglu et al^18^ (2006) evaluated the effects of different solutions on the tensile bond strengths of different soft liners. Fifty Dent was one of the cleansing agents that was evaluated. Based on the results, the bond strengths of soft liners stored in distilled water and Fifty Dent were almost similar, consistent with the results of the effect of Corega and Polident on tensile strength.

Gramipanah et al^19^ (2013) evaluated the effects of Corega and 2.5% sodium hypochlorite on the tensile bond strength of Mollosil , GC, Acropars and Molloplast B soft liners. According to the results of this study the type of the soft liner had a significant effect on the tensile bond strength; however, the type of the solution had no significant effect on the tensile bond strength of the samples. A lack of significant effect of cleansing agents on the soft liner in that study might be attributed to small sample size in that study, causing the differences in the results of the present study and that study.

Brozek et al^14^ (2011) evaluated the release of chemical components from the soft liners after immersion in different cleansing agents and reported that more components were released from all kinds of soft liners after immersion in sodium hypochlorite compared to immersion in Corega. Therefore, it might be claimed that loss of chemical components and plasticizers decrease the bond strength at bonded interfaces. These results might explain the greater decrease in bond strength in the present study after immersion in sodium hypochlorite solution.

In the present study, the samples immersed in Corega and distilled water predominantly exhibited cohesive failures and the samples immersed in sodium hypochlorite solution predominantly exhibited adhesive failures, possibly indicating that the decrease in bond strength affected the failure mode that was adhesive, consistent with the results of a study by Renata et al.^2,20^

Several studies, too, have evaluated the shear bond strength of soft liners.^21,22^ In a study by Satyanageshwar et al,^23^ the effect of saliva on shear bond strength of different soft liners to heat-cured denture bases was evaluated at 0-, 7- and 14-day intervals. The results showed that the shear bond strength decreased with an increase in immersion time.

## Conclusion

The results of the present study on the effect of cleansing solutions on the bond strength of soft liners to denture bases showed that immersion of soft liners in Corega will result in longevity of soft liners compared to immersion in sodium hypochlorite solution and sodium hypochlorite solution significantly decreased the tensile and shear bond strengths compared to the control and Corega groups.

## Acknowledgments

This paper was written based on the thesis submitted by Dr. Taghizadeh under the supervision of Dr. Mahboub.

## Authors’ contributions

FM was responsible for the concept of the study and FS and FP contributed to the study design.VG, YK and MT carried out the study procedures. MT drafted the manuscript. All the authors revised the manuscript, read and approved the final paper.

## Funding

This study was supported and funded by the Dental and Periodontal Research Center of Faculty of Dentistry, Tabriz University of Medical Sciences.

## Competing interests

The authors declare no competing interests with regards to the authorship and/or publication of this article.

## Ethics approval

Not applicable.
